# How researchers can translate health evidence into books for children

**DOI:** 10.1093/heapro/daae035

**Published:** 2024-05-09

**Authors:** Erin I Walsh, Ginny M Sargent, Laura Gooyers, Jessica Masters, Karima Laachir, Sotiris Vardoulakis

**Affiliations:** PHXchange (Population Health Exchange), National Centre for Epidemiology and Population Health, Australian National University, 62 Mills Rd, Acton 2601, Canberra, ACT, Australia; PHXchange (Population Health Exchange), National Centre for Epidemiology and Population Health, Australian National University, 62 Mills Rd, Acton 2601, Canberra, ACT, Australia; Australian Child and Adolescent Trauma, Loss & Grief Network, Australian National University, Building 4 Level 2 The Canberra Hospital Yamba Drive, Garran 2605, Canberra, ACT, Australia; Australian Child and Adolescent Trauma, Loss & Grief Network, Australian National University, Building 4 Level 2 The Canberra Hospital Yamba Drive, Garran 2605, Canberra, ACT, Australia; School of Literature, Arts and Media, University of Sydney, John Woolley Building, A20 Science Rd, Camperdown 2050, Sydney, NSW, Australia; Centre for Arab and Islamic Studies, Australian National University, 127 Ellery Cres, Acton 2601, Canberra, ACT, Australia; Healthy Environments And Lives (HEAL) National Research Network, Australia; National Centre for Epidemiology and Population Health, Australian National University, 62 Mills Rd, Acton 2601, Canberra, ACT, Australia

**Keywords:** knowledge translation, public health messaging, health promotion messages, population health communication, vulnerable populations, emerging health threats, communicating with children, air quality, bushfires

## Abstract

The health promotion literature that considers how scientific evidence can be effectively communicated tends to focus on evaluating the effectiveness of communication materials. This has resulted in a knowledge gap regarding effective knowledge translation processes. This study explores the process, reasoning and practices for developing books for children that incorporate evidence-based information to aid understanding of scientific evidence about health and environmental or natural disasters. This study is informed by a systematic review of the literature combined with responses to an email interview with authors of books for children. Nine published studies were included in the systematic review. Twenty-two authors responded to the email survey (25% response rate, following 86 invitations). We report seven key findings to guide the development of health-promoting books for children: (i) understand the needs and expectations of the audience, (ii) articulate the topic and research evidence, (iii) assemble a team with a mix of content knowledge and creative expertise, (iv) format should be chosen to suit the user group and guided by the creative team, (v) early testing with children and their support system is crucial, (vi) develop a dissemination strategy to reach the user group and (vii) engage in reflexivity through evaluation of effectiveness of messaging. The current investigation can guide the process, reasoning and practice of developing books for children that incorporate evidence about health and environmental disasters.

## Graphical Abstract

**Graphical Abstract:**
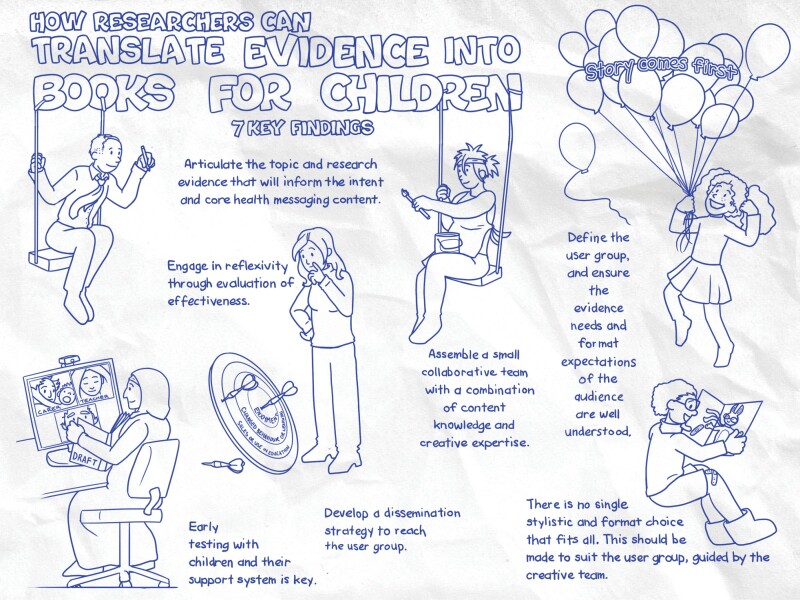

**Contribution to Health Promotion**

- We need to know more about how communication materials for effective health promotion can be developed.
- We explored the literature and interviewed practitioners to address this knowledge gap.
- We report a useful evidence base for developing books for children that incorporate evidence about health and environmental disasters.

## BACKGROUND

There is a substantial amount of evidence regarding short- and long-term risks to health, how to protect health and well-being, and more is generated every day (Klerings *et al*., 2015; Ghasemi *et al.*, 2023). Still, the majority of this evidence does not reach end users (Chalmers and Glasziou, 2009). The development of communication materials that are accessible and meet the needs of stakeholders is an important aspect of health promotion and knowledge translation (Macdonald, 2003; Brown *et al*., 2020). However, the effective translation of research findings into outputs that will guide policy, clinical practice and individual behaviour is a challenging process (Movsisyan *et al*., 2019; Kizer, 2020).

A scholarly focus on studying the effectiveness of knowledge translation outputs has resulted in a troublesome knowledge gap regarding effective knowledge translation processes (Chalmers and Glasziou, 2009). The translation research literature, describing how evidence can be effectively communicated, is even less well established when it comes to health messaging for the public (LaRocca *et al*., 2012). Existing frameworks have the potential to guide health knowledge translation, but using implementation science terms (per Nilsen, 2020), there is ample application of evaluation frameworks (which determine metrics and assessments of success) and insufficient application of process frameworks (which clarify determinants to achieve implementation success). A rare example of the latter is Jacobson *et al*. (2003)’s framework for understanding knowledge user context, which has been applied to knowledge exchange in public health (Armstrong *et al*., 2006).

The extent of public health messaging required during the Coronavirus disease pandemic has led to substantial innovation in health communication and highlighted particular demand for high-quality health communication that can reach people who are not well served by conventional public health messaging (Kizer, 2020; Wild *et al*., 2021). Children are one such group (Reid *et al*., 2017). Appropriately targeted psychoeducational and pragmatic preparedness can promote resilience in children (Peek, 2008). The value of knowledge translation approaches to support child health is recognized, but rigorous processes are underdeveloped, particularly in post-disaster recovery (Commers *et al*., 2014; Albrecht *et al*., 2017; Reid *et al*., 2017).

A major avenue for communicating with children is books. Books for children provide straightforward answers to most of the key knowledge translation considerations raised by Jacobson *et al.* (2003): they are familiar; an appropriate written format for children capable of meeting the reader’s preferences; allow a family-centred knowledge translation approach that invites parent/child interaction and engagement, more so than other candidate mediums for communicating health information, such as video games (Connell *et al*., 2015; Reid *et al*., 2017).

Children’s books with health themes existed prior to the 19th century, before health education was codified as a discipline (Ubbes and Ausherman, 2018). There is an established literature describing the psychology and educational utility of children’s picture books (Nodelman, 1988). This is largely separate from the body of research on educating children on the topic of environment and health (e.g. Maibach and Parrott, 1995; Midtbust *et al*., 2018). Both literatures pay the most attention to the characterization, and evaluation of completed communication materials and synthesis between these literatures is lacking. Solely focusing on finished works, without discourse describing their inception and design, provides only marginal evidence that might guide the development of books for children that effectively translate evidence-based messages.

Health literacy, and related concepts of environmental literacy, environmental health literacy, science literacy and information literacy focus attention on the reader’s capacity to engage with information, which provides some guidance for the creation of health messaging in general (Nutbeam, 2008), and books for children containing health messaging (e.g. as in Tarver *et al*., 2016). Still, there is substantial variation in how health literacy is applied in the design and implementation of child health interventions, and how learning and behavioural outcomes are evaluated across a wide range of health topics (Naccarella and Guo, 2022). An important topic for such books, and the focus of the current study, is the communication of messages about health and natural disasters.

An estimated 175 million children will be affected by natural disasters attributable to climate change, such as bushfires (Codreanu *et al.*, 2014). There is substantial heterogeneity in how children may react to natural disasters, explained in part by developmental level, mental health history, culture, biological and environmental differences (Aptekar and Boore, 1990; Engle *et al*., 1996; Li *et al.*, 2013; Taylor and Peace, 2015). In recent years, large-scale fires (also referred to as bushfires and wildfires) have caused damage to human, ecosystem and planetary health (Vardoulakis *et al*., 2020). A vivid example was the season of extensive and intense bushfires experienced in Eastern Australia during the ‘Black Summer’ of 2019/2020. These affected, directly or indirectly, around 10 million Australians (Borchers Arriagada *et al*., 2020). These fires generated an over 5 million km^2^ smoke plume that persisted for over 3 months and profoundly impacted the daily lives of Australians even in areas not directly burned (Khaykin *et al.*, 2020; UN, 2020; Williamson *et al*., 2022). There is evidence that the health and well-being effects were particularly pronounced in children, and these effects are magnified in vulnerable and marginalized communities (Curtin *et al*., 2020; Milton and White, 2020; Bessell, 2021). Because children are at particular risk of the harmful impacts of climate change, it is important that knowledge translation materials are developed to better target this population with public health messaging.

Due to an overwhelming focus on the evaluation of completed materials in the existing literature, there is currently a knowledge gap surrounding the process, reasoning and practice of developing books for children that incorporate evidence about health and environmental disasters. Contributing to filling this gap in knowledge translation research, this study uses a multi-method approach to assist in the timely production of impactful knowledge translation materials suitable for children. This review will include all health and natural disaster information, with a particular interest in materials relevant to bushfires and bushfire smoke given their ongoing relevance to children in Australia, and worldwide.

## METHODS

Situating our approach within Rychetnik *et al.* (2012)’s integrated framework of knowledge translation to support evidence-based practice, this multi-method approach collates evidence of current practice for the development of books for children, in order to identify solutions to the problem of communicating information about natural disasters and health to children. Insights were sought from the scholarly literature, and authors with direct relevant experience in the publication industry in developing books for children about health and/or natural disasters. Reflecting the possibility that relevant knowledge may not flow freely between academia and industry noted in Moncaster *et al.* (2010), this dual approach was taken to combine scholarly research with real-world experience. Our synthesis has been heavily informed by Jacobson *et al.* (2003)’s framework for knowledge translation, focussing on the elements of defining the user group and understanding their needs, articulating the evidence-based key messages, assembling a team and reaching the user group.

### Literature review

**Fig. 1:**
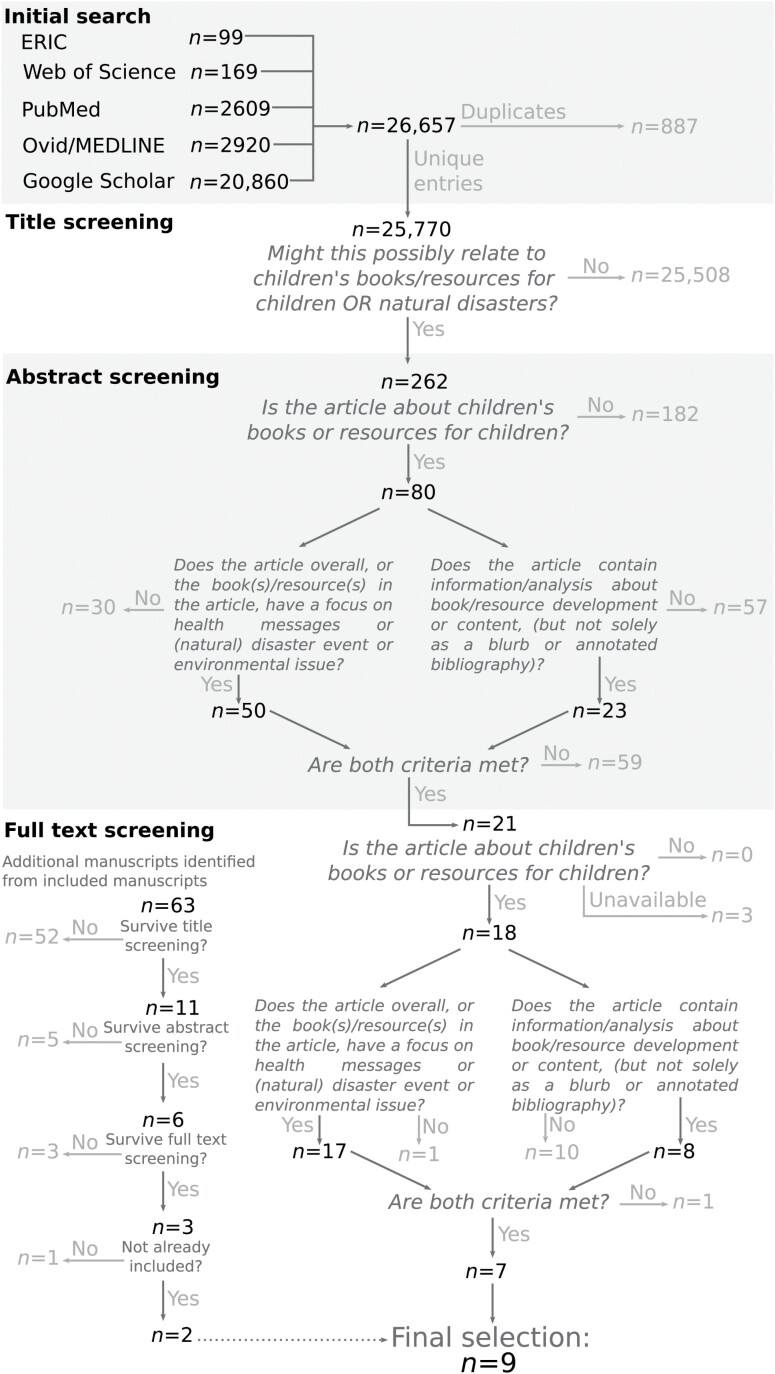
Systematic literature review protocol, including specific search terms and screening criterion.

The systematic literature review protocol, including specific search terms and screening criterion, is outlined in Figure 1. Broadly, the approach was to search for iterations of the term ‘children’s picture book’, and ‘health’ or ‘disaster’ across health-related (Web of Science, PubMed, Ovid/MEDLINE), generalist (Google Scholar), and educational (Education Resources Information Center) scholarly search engines, with all of their constituent databases included in the search. (Web of Science includes six databases: Science Citation Index Expanded, Social Sciences Citation Index, Arts & Humanities Citation Index, Emerging Sources Citation Index, Book Citation Index. Ovid/MEDLINE included Journals@Ovid, Books@Ovid, APA PsycArticles Full Text, APA PsycInfo, CAB Abstracts and MEDLINE.) Search terms were established a priori, and are as follows:

The search was carried out on 2 September 2020 (ERIC database search was included following peer review, and the search was carried out on 8 November 2023.) with the following strings: (‘book for children’ OR ‘children’s book’ OR ‘children’s books’ OR ‘comic book’ OR ‘comic-book’ OR ‘comic books’ OR ‘picture book’ OR ‘picture-book’ OR ‘picture books’ OR ‘story book’ OR ‘story books’ OR ‘story-book’ OR ‘storybook’) AND (health OR disaster).

Title screening was carried out by one author (E.W.). The inclusion/exclusion criteria are included in Figure 1 and expanded upon in Supplementary Table 1. Abstract screening was carried out by two authors (L.G. and J.M.). Full-text screening was carried out by one author (E.W.). Additional candidate manuscripts were identified as those citing or cited by included manuscripts and screened using the same protocol. Data extraction focused on content of disaster or health messaging, child target age, reported intention of the book, source of information used within the book, authorial team, book format, stylistic choices, process of drafting and refinement, method of dissemination and evaluation of effectiveness and impact.

### Author interviews

The authors of books for children relating to natural disasters and health messages were identified via the presence of their work in two largest online databases of books: Google Books and Amazon Books (Australia). The same search string used for the systematic review (see Figure 1) was entered into both databases. For Amazon Books, the additional steps of limiting results to the ‘Children’s literature’ department and tagged with ‘picture book’ were taken to focus the search. Author contact details were obtained via inspection of the digital copies of these books and/or Google searches of author names. All 86 authors whose contact details were found were approached via email or web form and invited to participate in this study and provided an information sheet. The authors then responded via email or teleconference. Questions in full are listed in Supplementary Table 2. All interviews were undertaken in English. Responses were qualitatively synthesized by E.W., by the application of inductive qualitative content analysis as described in Elo and Kyngäs (2008). Briefly, this approach is characterized by three phases: preparation (where materials are gathered and the data as a whole are reviewed and made sense of), organization (where the data are coded, grouped into meaningful categories and abstracted) and reporting. Reporting was structured to reflect thematic groupings established following review of included manuscripts.

## RESULTS

### Literature review

The systematic literature review identified nine manuscripts. Four of these described books focused on natural disasters including typhoons, hurricanes and storms (Sharpe and Izadkhah, 2014; Marvida and Tibayan, 2019; McKeon, 2020; Solfiah *et al*., 2020), and five related to health messaging including sun safety and juvenile idiopathic arthritis (Thornton and Piacquadio, 1996; McDermott and Lowe, 2003; Armstrong, 2012; Egert *et al*., 2017; Robinson *et al.*, 2017). None of the identified manuscripts described the development of books for children relating specifically to bushfires or bushfire smoke.

### Author interviews

The *n* = 483 results from Google Books and *n* = 1164 results from Amazon Books were screened by title and cover illustration by one author (E.W.) for inclusion. This identified *n* = 215 books for children, which included pictures in some form, and themes relating to health or natural disasters. Of the 86 authors contacted, 22 participated (25% response rate). One teleconference interview was conducted; the remainder of responses were from email correspondence. While demographic information was not collected, broadly, this sample included a mix of new and established authors, most of whom resided in the USA or the UK. Respondent names, the book that led to their inclusion in this study, and links to their websites and/or lists of their work can be found in Supplementary Table 3.

### Synthesis

#### Defining the user group—understanding the evidence needs and format expectations of the audience

All manuscripts indicated their primary target audience was children, with age ranges of 2–3 years (as in McDermott and Lowe, 2003), 2–6 years (as in Robinson *et al*., 2017), 5–6 years (as in Solfiah *et al*., 2020), 5–8 years (as in Armstrong, 2012), 8–9 years (as in Thornton and Piacquadio, 1996), and the remainder not specifying a specific age range (Egert *et al.*, 2017; Marvida and Tibayan, 2019; McKeon, 2020). Most authors interviewed who discussed the target age group noted the age ranges of their works were typically dictated by their publisher.

Most of the manuscripts did not draw from educational, knowledge exchange or other scholarly theory to understand their audience. Robinson *et al.* (2017) applied theory focussing on individual processes, and Armstrong (2012) and Solfiah *et al.* (2020) drew from theory focussing on interaction between individuals. Consultation with carers, clinicians and educators was used to identify needs and knowledge gaps (as in Robinson *et al*., 2017), appropriate examples of extant media (as in Sharpe and Izadkhah, 2014) and expert insight on both substantive content and best practice for developing the book (as in Marvida and Tibayan, 2019; McKeon, 2020).

This was similar in author interviews: seven reported consulting with educators and carers. Several others noted that they drew from their own relevant backgrounds, typically in education or carer roles for children. There was substantial consensus that children tend to be more insightful and resilient than adults give them credit for, and thus the importance of respecting the audience. A common theme across interviews was that it is a mistake to think of ‘children’ as a singular group. Even within the same age bracket, children have different life experiences and vary on individual traits such as reading aptitude, personality, emotional resilience, aesthetic preferences, and cultural and linguistic backgrounds.

In the manuscripts, parents were typically described in terms of their role as curator, that is procuring and presenting the book to the child (as in Solfiah*et al*., 2020). Robinson *et al.* (2017) note that parents reading books with health messages aloud with their children has the triple benefit of promoting parent–child bonding, improving the child’s literacy and instilling and reinforcing healthy behaviours in both the parent and the child. In interviews, four authors indicated that their writing accounts for the possibility that parents may be reading the books out loud to their children, thus parents become part of their audience. This led to consideration of including some elements for the parents as well as children, and the role of parental interpretation for children who may be too young to understand the content as written.

In terms of audience, I think of picture books as being for families. They’re for the young kids, but the older kids can also sit in, the parents are probably reading it aloud. So, it’s kind of a family affair. BH

#### Articulating the topic and research evidence that will inform the intent and core health messaging

Present in six manuscripts, the most commonly reported intention of the books under development was to impart knowledge to children about a topic that could affect the reader’s health (McDermott and Lowe, 2003; Armstrong, 2012; Sharpe and Izadkhah, 2014; Egert *et al*., 2017; Robinson *et al*., 2017; Solfiah *et al*., 2020).

Among book authors, the most common source of motivation for choosing a particular topic, and inspiration for specific stories, was personal experiences with the topic (*n* = 6), particularly when authors had witnessed first hand the impact of that experience on their children or children in the affected community (*n* = 4).

A common theme across authors was the hope that their books might provide a novel perspective, experience or information. Some were explicit about their intent for the book to help children deal with difficulty in instrumental (e.g. preparation of a storm kit) or emotional (e.g. understanding it is OK and normal to be scared) domains.

Above all, I wanted to equip my readers for tough emotional times that might lie ahead for them. CB

Though implicit in the development process, only four manuscripts explicitly highlighted their intent to engage children, on the logic that knowledge transfer and messaging can only be successful if a child is actively attending to the book’s contents (McDermott and Lowe, 2003; Armstrong, 2012; Solfiah *et al*., 2020). In contrast, almost all authors indicated that they intended for children to engage with the book and expressed an extremely wide range of ways they manifested this intent in their work.

Where nonfiction is concerned, my overall aim has been to approach a topic in a way that will be engaging as well as informative. SK

Within manuscripts, the source of health-relevant content was almost always provided by one or more domain-specific expert within the co-author team, with only three drawing from the scholarly literature to inform topics that would be conveyed by the book (Robinson *et al*., 2017; Marvida and Tibayan, 2019; McKeon, 2020). In interviews, authors indicated they did not draw from formal educational, knowledge exchange or other scholarly theory to inform the development of their books, though just under a third (*n* = 8) reported consulting the scholarly literature (i.e., peer-reviewed journals or textbooks) to ‘fact check’ the substantive information in their books.

#### Collaborative small team with content knowledge and creative expertise

The majority of books for children described in manuscripts were both written and illustrated by the same individuals. Only McDermott and Lowe (2003) indicated working with a professional writer, and Armstrong (2012) and Egert *et al.* (2017) indicated working with an illustrator outside of the manuscript authorship team. Author interviews revealed examples of more collaborative teams, with very clearly delimited roles. Seven of the authors illustrated their books themselves, and the remaining 15 worked with an illustrator.

The editor (working for the publishing house) has the expertise to know what sells and chooses the illustrator based on their knowledge, their available illustrators and their vision for the book (after conversations with the author, of course). Then the illustrator reads the book with fresh eyes and sees how his particular style can work with what the editor is asking for. HT

Regardless of how closely these team members worked, most authors raised the benefits of multiple perspectives for the creation of the book and indicated that these benefits relied on trust within the team. Trust from the publisher or any other stakeholder was also seen as important to allow creative freedom.

Speaking from experience, I can tell you that nothing is more likely to discourage a creative endeavour than committee meetings, red tape and restrictions. CG

#### Stylistic choices for engagement and content delivery

Book format is a key part of engaging the audience and includes the size, shape, pagination, words and illustration. Few manuscripts specified the number of pages in the final book, with Robinson *et al.* (2017) indicating 13 pages and Armstrong (2012) indicating 31 pages. The most consistent text format choice was the use of large, sparse text (Armstrong, 2012; Robinson *et al.*, 2017; Marvida and Tibayan, 2019). Marvida and Tibayan (2019) specified the additional consideration of easily read fonts, such as Hallo Sans. Among author interviews, technicalities such as number of words per page were not discussed.

Techniques discussed to engage children included intermixing text and imagery; applying accessible but playful language (e.g. through rhyming or onomatopoeia); crafting narratives resembling child-friendly stage plays creating characters and situations that sparked empathy and perspective-taking; and wherever possible including humour targeted at both child and adult readers. Many authors indicated visuals should be striking, vibrant and attention-grabbing, and their reflection focused on narrative and stylistic (rather than aesthetic) methods of achieving this.

Stylistic choices in text included rhyming onomatopoeia, language or wordplay, structure deriving from poetry and purposeful choice of the shortest and least ambiguous words possible. Several authors outlined strategies for including additional informational content, largely by the separation of text into integral and ‘optional’ elements, such as footnotes, side bars and text-only pages at the end of the book. Outside of these elements, a common theme was that the text should match well with the visuals thematically and spatially.

[…] we wanted the movement and flow of the text to merge with the pictures and create a work of art that children could “feel” and be drawn in to. AW

Several manuscripts included the rationale that the characters in the book should be easy for child readers to identify with, and model desired behaviours or outcomes in the child and/or parent (Thornton and Piacquadio, 1996; McDermott and Lowe, 2003; Armstrong 2012; Robinson *et al*., 2017). While the reason for using anthropomorphic animals was often not discussed, they were the most common protagonists (as in McDermott and Lowe, 2003; Armstrong, 2012; McKeon, 2020; Solfiah *et al*., 2020).

One author was unconvinced regarding the purported benefits of using anthropomorphized animals, but the rest of the authors indicated support for this practice. Two key reasons for the use of anthropomorphized animals emerged: as an aesthetic tool to hold children’s attention, and to create a character all children could identify with regardless of their ethnicity, race or culture. One author further indicated that the use of animals provided a ‘safe’ distance between the events depicted, and the child’s sense of self.

It was important to make the story just scary enough to hold readers’ attention and make them feel some anxiety, without being overly frightening and creating trauma. Children relate well to animals, so I think using animals in the main roles is a technique that works here. ES

When describing the narrative, book authors cautioned against being pedantic, didactic, ‘telling’ facts and creating something more akin to a manual than a story. Several authors pointed out that children do not need strong prompting to learn due to their natural curiosity, which they naturally want to learn, so consequently subtle educational elements can be highly effective.

### Early testing with children and their support system

Once a first draft or ‘prototype’ book was developed, approximately half of the manuscripts identified described some form of iterative pilot testing. This process was universally qualitative, consisting of focus groups or interviews with educators, clinicians or carers who worked with the target children (as in Armstrong, 2012; Robinson *et al*., 2017), or with educators, clinicians or carers and children themselves (as in McDermott and Lowe, 2003; Solfiah *et al*., 2020).

#### Dissemination strategy to reach user group

Before a book may reach a parent, educator or child, it must be published. The most commonly reported method in manuscripts was self-publication (as in Sharpe and Izadkhah, 2014; Marvida and Tibayan, 2019; McKeon, 2020). McDermott and Lowe (2003) and Robinson *et al.* (2017) provided a copy of the final book to the study cohort, with Robinson *et al*. (2017) additionally donating copies of their book to local paediatric clinical settings. Commentary on the broader context of the publication process was largely absent.

Conversely, for authors, the end point of dissemination dictated the likelihood of beginning the development of a book, and many aspects of its content and focus. Several authors raised the broader context of their books, both with contemporary libraries and in terms of the history of the children’s book publishing industry. Many authors saw their work as adding to a broader body of books on a particular topic or expressing particular themes.

Young readers are not monolithic. […] Multiple books—rather than a single publication […]—may well be needed in order to reach a significant number of children, but I believe that all such books should speak to this audience honestly and with respect for their intelligence. CB

#### Reflexivity through evaluation of effectiveness

Five manuscripts described evaluations of the completed books as part of their development, taking place after the books were distributed (noting only Egert *et al*., 2017 reported that the book was formally published). This was achieved via quantitative (as in Marvida and Tibayan, 2019), qualitative (as in Armstrong, 2012; Sharpe and Izadkhah, 2014) or both quantitative and qualitative (as in McDermott and Lowe, 2003; Robinson *et al*., 2017) approaches. Key informants were universally the source of feedback on the impact of the completed book, including parents (as in McDermott and Lowe, 2003; Armstrong, 2012; Robinson *et al*., 2017; Marvida and Tibayan, 2019) and educators (as in Armstrong, 2012; Sharpe and Izadkhah, 2014).

Measures to evaluate the quality or fitness-for-purpose of books were heterogeneous, ranging from Marvida and Tibayan (2019)’s Likert axes of attractiveness, comprehensibility, persuasion, self-involvement and acceptability, to qualitative parent report of how much a child enjoyed the book (as in McDermott and Lowe, 2003). The most common measure that could be taken as a proxy for the quality of the book was the frequency of its use by the child (as in McDermott and Lowe, 2003; Sharpe and Izadkhah, 2014; Robinson *et al*., 2017).

The effectiveness of the developed books on children was characterized by a variety of indicators, including information retention (as in Sharpe and Izadkhah, 2014), behaviour change (as in Robinson *et al*., 2017), change in emotions relating to the topic depicted and discussions instigated by children with peers or others about the topic depicted (as in Sharpe and Izadkhah, 2014). McDermott and Lowe (2003) provided free copies of their book and investigated perceived value by asking how much the parent would be willing to spend to purchase the book had it not been provided.

The author’s reflections on the success of their books could be broadly categorized as completion of the book, sales/financial success and, most importantly, impact. Inclusion in educational reading lists or curriculum (at the state or national level) or presence in libraries were widely seen as useful proxies of impact, as they speak to reach and wide appeal. Some authors also mentioned critical reviews online or elsewhere were useful, but most indicated that feedback from parents, teachers and children was a more genuine reflection of the impact of their work.

For me, success with this book means knowing that readers connected with it. Hearing from readers that they liked the book and that it affected them is the main benchmark of success for me. When a child tells me that they loved the book, or that they know all the characters by name, that means everything. ES

## DISCUSSION

This study has brought together scholarly publication and insights from authors of books for children to provide detailed insight into the process, reasoning and practises for developing books for children about health and/or natural disasters. The approaches described in the scholarly literature tended towards an atomistic view, where storytelling and engagement are framed as tools for reaching the goal of knowledge transfer and behaviour change, which could consequently be quantified. Conversely, authors of books for children tended to take a more holistic view, where engagement, knowledge transfer, storytelling and impact on children are interwoven, rendering them less tangible and thus difficult to quantify. Synthesis of the research translation practice of academics with the book development practice of authors is a solid foundation for the future development of books for children about health and environmental disasters.

In contrast to focussing on how text, colour or form should best be used (e.g. as in Reuter, 2007), there was no clear consensus in our current investigation regarding specific elements underlying ‘good’ design or aesthetics in books for children. Academics focussed on granular elements of aesthetics (e.g. shape, colour, use of one- or two-dimensional art), while authors focussed on the holistic elements of aesthetics (e.g. aiming for vibrancy and attracting attention). This latter approach aligns with work in related fields that indicates that any individual’s engagement with visuals and information arises from an admixture of aesthetics, personal history and context (e.g. superhero design in Demirbilek and Sener 2004 and health information for an adult audience in Marshall and Williams 2006).

Both academics and authors broadly recommended the use of animal characters and/or anthropomorphizing animals as a tool for allowing a wide range of children to identify with characters. Children identify with the thoughts, feelings and more readily characterize the internal state of animal characters (both realistic and anthropomorphized) than they do for comparable human characters (Russell and Cain, 2020). This is also reminiscent of the use of cartoons and puppets to teach children about complex and potentially distressing information in fields such as healthcare (e.g. Tilbrook *et al*., 2017).

The most striking and educative difference between academic process and authorial process is the composition of the team creating the book. Most authors reported a team of at least two individuals (editor, writer/illustrator) providing multiple perspectives from specialized viewpoints and working with varying degrees of closeness to in create a single output. This reflects a wider literature highlighting the importance of the editorial role in all forms of book production (Gosney, 2017; Reeve, 2018). Taken together, it is clear that high trust and division of labour between one (or more) individuals serving as editors and one (or more) individuals focussing on the creative aspects of the book (text and illustration) are beneficial for retaining both conceptual focus and imagination.

Co-design with carers, clinicians, educators and experts with in-area knowledge was common in both academic process and author process of book development. Co-design can be extremely productive, but also difficult to achieve, and costly in terms of time and people (Lee, 2008; Whicher and Crick, 2019). Nonetheless, there are emerging paradigms for overcoming the pragmatic barriers of conducting co-design with children (e.g. Durl *et al.*, 2017). Combining the approaches taken by academics and authors, the importance of co-design with carers, clinicians, educators, experts and especially children is reflected in the related literature (Alhumaidan *et al*., 2018; Södergren and Van Mechelen, 2019). This suggests that co-design is important during the conceptualization, development (as typically employed by academics) and evaluation (as typically employed by authors) of a book for children.

In the specific case of public health and health promotion, there is a need to directly include the voices of children themselves in the design and dissemination of knowledge translation materials for health and/or natural disasters for themselves, their peers and the broader community. This approach exemplified by the work of Maggie Mort *et al*., who have engaged in a program of research over the past decade to source and amplify children’s voices in natural disaster preparedness and recovery (see Mort *et al*., 2016; Mort and Rodriguez-Giralt, 2020). Mort *et al*. have shown how children’s perspectives from interviews, writing and drawing can empower their resilience to floods, and also meaningfully change social structures surrounding floods such as insurance company policy (Williams *et al*., 2017; Mort *et al*., 2018).

A theme almost entirely absent in the academic discourse was the author’s focus on children’s strengths: curiosity, empathy and resilience. These topics are covered within the scholarly literature in detail elsewhere (e.g. Li *et al*., 2013; Engle *et al*., 1996), but this was disconnected from how books were developed. This reflected a broader tendency within academic discourse to overlook the individual characteristics of children (with the exception of age group), with a notable lack of investigation of individual characteristics that may both make the child more vulnerable to the health impacts of environmental disasters and impact how they engage with the book. This should be followed up in future research, given the importance of individual characteristics for health promotion in adult samples (Golden and Earp, 2012).

A related theme almost absent was cultural awareness and symbolism. Although focused on literature published and communicated in English, this review indicates a lack of culturally appropriate and linguistically diverse stories (e.g. translated into multiple languages) that reflect the nuances, complexity and needs of children cosmopolitan communities affected by natural disasters. The wider landscape of children’s books includes many examples of cultural diversity (e.g. see the listings of annual multicultural children’s book day, https://multiculturalchildrensbookday.com/). Given the potentially disproportional impact of natural disasters on Culturally and Linguistically Diverse (CALD) communities, and the increased need for age-appropriate health messaging in this context (Ogie and Pradhan, 2019; Ng *et al*., 2021). Cultural context is important to inform both the development and surrounding research/evaluation of health promotion communications and interventions (Dickerson *et al*., 2020). The lack of culturally appropriate and linguistically diverse children’s stories on natural disasters and health is identified as a significant gap in this literature on the development of books for children.

It is important for researchers to build an understanding of the publication industry. This understanding can improve their processes for the development of books, and enhance the likelihood their work will be picked up and disseminated by publishers (Moncaster *et al*., 2010), or featured on curated lists such as ‘Reading Well’ (https://reading-well.org.uk/books/books-on-prescription/children). Relatedly, librarians, educators and other professionals who work with children understand the landscape of what is available, what is needed and what is likely to appeal to children. This does not always map onto children’s preferences (e.g. Beach 2015), but importantly, their judgement curates which book children have access to in libraries and the classroom.

Parental choice of books, and how they share the books with the child (e.g. reading together, promoting discussion), can have long-term impacts on child development (Daniels *et al*., 2021). Parental or carer involvement is a key link in the chain between evidence and a child’s understanding of information about natural disasters and consequent health promotion behaviour (Habib *et al*., 2010; Midtbust *et al*., 2018). Like adults, children need to be prepared to respond to natural disasters and other challenges to their health by altering their daily behaviour (e.g. staying indoors or wearing a facemask) and recovering from potentially traumatic experiences (e.g. smoke inhalation or relocation). Unlike adults, children cannot make larger decisions (e.g. relocation) for themselves, but can be agentic in raising and complying with actions that protect themselves (e.g. staying indoors). Reflecting on the emotional effects of natural disasters on children, Aptekar and Boore (1990) state ‘Perhaps in no other human experience does the weave between child and parent intertwine so strongly’ (p. 77). The parent’s knowledge, preparedness and behaviour forms a template for the child’s behaviour (Midtbust *et al*., 2018). From a health literacy perspective, materials suitable for children can also assist in informing parental or caregivers (Tarver *et al*., 2016). In health promotion specifically, there is evidence that parental involvement is an important link between information provision and behaviour change in young people (Perry *et al*., 1989).

This hybrid systematic literature review has some notable strengths and limitations. The key limitation was inclusion only of English-language literature, and authors publishing in English. To avoid missing information, we allowed all sources to self-identify what ‘child’ means, but this did result in inability to comment on the different needs of a 2 year old vs. 16 year old.

The greatest strength of this review was the hybrid approach. By taking the novel approach of comparing and combining the structure of insight from the literature, and the qualitative depth of insight from author interviews, this study is able to synthesize evidence- and practice-based understanding that can enhance the future development of books for children about health and/or natural disasters. Further research would be enriched by the inclusion of perspectives from other roles involved in the creation and distribution of books for children, including illustrators, publishers and librarians.

Though the initial search scope was broad, few manuscripts met the inclusion criterion because the scholarly literature is more focussed on the evaluation of existing books, rather than documenting the process of how the books were developed. This is resonant with the wider problem of tacit information loss well characterized in the organizational literature (e.g. Martins and Martins, 2011) and noted within many domains within the scholarly literature (Chalmers and Glasziou, 2009). Much could be gained from following the example of the rich literature surrounding design thinking (e.g. Beckman and Barry, 2007), which focuses as much on process as it does on outcomes. Similarly, the current review is limited by its focus on books as print media, as noted elsewhere, communications are most effective in promoting health when utilized alongside other interventions (James *et al*., 2005). The review did not actively exclude e-books, but the search strategy focused on vendors of physical books. Further research could use resources such as the Children’s Picture Book Database at Miami University (https://dlp.lib.miamioh.edu/picturebook/), as used in Ubbes and Ausherman (2018), and e-book storefronts such as https://childrensebooks.com.au/ could be used to identify authors of relevant e-books for children.

## CONCLUSION

This review synthesizes scholarly and applied insights into the process, reasoning and practises for developing books for children about health and/or environmental or natural disasters. Our work reveals seven aspects to guide how researchers and non-researchers can translate evidence into books for children about health and/or natural disasters:

1. Define the user group and ensure the evidence needs and format expectations of the audience are well understood.
2. Articulate the topic and research evidence that will inform the intent and core health messaging content.
3. Assemble a small collaborative team with a combination of content knowledge and creative expertise.
4. There is no single stylistic and format choice that fits all—this should be made to suit the user group, guided by the creative team.
5. Early testing with children and their support system is key.
6. Develop a dissemination strategy to reach the user group.
7. Engage in reflexivity through evaluation of effectiveness.

## Supplementary Material

daae035_suppl_Supplementary_Tables_1-3_Figures_S11-S17
