# Supplementary material for: How researchers can translate health evidence into books for children
Source: Health Promot Int. 2024 May 9;39(3):daae035. doi: 10.1093/heapro/daae035 (PMC11079950; doi:10.1093/heapro/daae035)
Supplement: daae035_suppl_Supplementary_Tables_1-3_Figures_S11-S17 [file daae035_suppl_supplementary_tables_1-3_figures_s11-s17.docx]

Supplementary table 1. Inclusion/exclusion criteria and further explanation

| Phase | Criterion | Definitions |
| --- | --- | --- |
| Title screening | Might this possibly relate to (a) children’s books/resources for children OR (b) natural disasters? | Resources for children: any written or visual form of information |
| Abstract screening | Is the article about  (a) Children’s books OR  (b) Resources for children? | Children’s books: any book described as a book for children, or listed in children’s books sections of libraries or bookstores |
| Abstract screening | Does the article overall, or the book(s)/resource(s) in the article have a focus on health messages or natural disaster event or environmental issue? AND  Does the article contain information/analysis about book/resource development or content (but not solely as a blurb or annotated bibliography)? | Health messages: any messages designed to raise awareness of health-related topics, or change behaviours relating to health  Natural disaster: an event such as a fire, flood, or earthquake that causes widespread damage or loss of life |
| Full text screening | Is the article about children’s books or resources for children? |  |
| Full text screening | Does the article overall, or the book(s)/resource(s) in the article have a focus on health messages or natural disaster event or environmental issue? AND  Does the article contain information/analysis about book/resource development or content (but not solely as a blurb or annotated bibliography)? | Environmental issue: the relationship between human activity and the environment |

Supplementary table 2. Questionnaire distributed to authors of children’s books via email

| Theme | Question text |
| --- | --- |
| Development | When you were putting the book together, was your work informed by…  - Work from scholarly theories (e.g. developmental phases or play theory)?  - Formal scientific information (from scholarly journals or textbooks)?  - Consultation with people like educators or psychologists?  - Were there any other source of inspiration or ideas that informed what went into the book?  - Did you work in a team (e.g. with an illustrator, or writer, publisher, or expert in the topic) when you made the final draft? If so, can you comment on any challenges and benefits of doing so? |
| Intent | In our review, some authors wanted their books to grab children’s attention, others want to teach them something, and others want the children to talk to their friends about what they have read. How did you hope your book would engage the children reading it? |
| Evaluation | In our review, some authors saw finishing the book as their end goal. Others looked at sales figures, and others interviewed teachers and children to try to understand the impact their book has had. What does “success” for your book mean to you? How did you measure it? |
| Open-ended | Is there anything else you think is important or would like to add about the development of books relating to natural disasters for children? |

Supplementary table 3. Full list of respondents, and their bibliography at the time of publication.

| Author name | Book communicating health information and/or regarding environmental disasters used to identify the author | Website |
| --- | --- | --- |
| Adam Wallace | Spark | <https://www.adam-wallace-books.com/> |
| Beth Bacon | COVID-19 Helpers | <https://bethbaconauthor.com/> |
| Bridget Heos | It's Getting Hot in Here: The Past, the Present, and the Future of Global Warming | <http://www.authorbridgetheos.com/> |
| Celia Godkin | Fire! The renewal of a forest | <https://www.celiagodkin.com/> |
| Chris Barton | All of a Sudden and Forever: Help and Healing after the Oklahoma City Bombing | <https://research.monash.edu/en/persons/chris-barton> |
| Emma Bland Smith | Odin, dog hero of the fires | <https://emmabsmith.com/> |
| Eric Arnold | Volcanoes!: Mountains of Fire (Step into Reading) | <https://www.penguinrandomhouse.com/authors/846/eric-arnold/> |
| Gerald Hausman | The Otter, the Spotted Frog & the Great Flood: A Creek Indian Story… Little Miracles | <http://www.geraldhausman.com/> |
| Heather Tekavec | Storm Is Coming | <https://www.tekavec.com/> |
| Janice Dean | Freddy the Frogcaster and the Flash Flood | <http://www.freddythefrogcaster.com/> |
| Jessee Johua Watson | Hope for Haiti | <http://www.jessewatson.com/home> |
| Matthew McElligott | Mad Scientist Academy: The Weather Disaster | <https://www.matthewmcelligott.com/newwebsite/> |
| Nancy Carlson | What If It Never Stops Raining? | <https://nancycarlson.com/> |
| Paul Bright | Bears in the Bed and the Great Big Storm | <https://paulbright.co.uk/> |
| Paulette Bourgeois | Franklin and the Thunderstorm … Franklin Gets Lost | <https://www.paulettebourgeois.ca/> |
| Petra brown | When the wind blew | <https://www.petrab.co.uk/> |
| Philip Steele | Volcanoes | <http://www.philipsteele.co.uk/> |
| Rheah Tregebov | The Big Storm | <http://rheatregebov.ca/> |
| Robert Neubecker | Little Smokey | <https://www.robertneubecker.com/> |
| Simon Adams | The Best Book of Volcanoes | <http://www.simon-adams.co.uk/> |
| Stephen Krensky | Lizzie Newton and the San Francisco Earthquake | <http://stephenkrensky.com/> |
| Tim McCanna | Watersong | <http://www.timmccanna.com/> |
